# Timing from admission to debridement, antibiotic, and implant retention (DAIR) affects mortality risk in total hip prosthetic joint infections

**DOI:** 10.5194/jbji-11-463-2026

**Published:** 2026-08-03

**Authors:** Jack Legler, Samuel Morgan, Paul Beaulé, Hesham Abdelbary, George Grammatopoulos, Simon Garceau

**Affiliations:** 1 Division of Orthopaedic Surgery, McGill University, 1650 Cedar Avenue, Montreal, Quebec H3G 1A4, Canada; 2 Division of Orthopaedic Surgery, The Ottawa Hospital, University of Ottawa, 501 Smyth Road, Ottawa, Ontario K1H 8L6, Canada; 3 Inflammation and Chronic Disease Program, The Ottawa Hospital Research Institute, 1053 Carling Avenue, Ottawa, Ontario K1Y 4E9, Canada; 4 Clinical Science and Translational Medicine Program, Faculty of Medicine, University of Ottawa, 451 Smyth Road, Ottawa, Ontario K1H 8M5, Canada

## Abstract

**Introduction**: Recent work in total knee arthroplasty (TKA) prosthetic joint infection (PJI) suggests that timing from hospital admission to DAIR (debridement, antibiotic, and implant retention) is a modifiable risk factor influencing treatment outcomes. The study assessed the impact of timing from admission to DAIR on clinical outcomes and treatment success in the total hip arthroplasty (THA) PJI population. **Methods**: A retrospective review was conducted at a specialized PJI tertiary referral centre. Patients who underwent DAIR for THA PJI between 2008 and 2021 with a minimum 2-year follow-up were included. The primary outcome was reoperation for recalcitrant PJI. Secondary outcomes included 90 d readmission, 90 d and 1-year mortality, and postoperative complications. Multivariate regression analysis identified factors associated with DAIR outcomes. **Results**: A total of 100 patients satisfied the inclusion criteria. The mean time from admission to DAIR was 46.4 ± 45.1 h, and 52.0 % required reoperation for recalcitrant PJI. Prolonged time from admission to DAIR was associated with increased 90 d mortality (odds ratio or OR: 1.02, CI (confidence interval) 95 %: 1.00–1.03, p= 0.04). Increasing age was associated with greater 1-year mortality (OR: 1.06, CI 95 %: 1.00–1.12, p= 0.049). McPherson host grade C compared to grade A was associated with both greater 1-year mortality (OR: 12.75, CI 95 %: 1.11–146.09, p= 0.04) and postoperative complications (OR: 7.59, CI 95 %: 1.22–47.08, p= 0.03). McPherson extremity grade II versus grade I (OR: 3.28, CI 95 %: 1.21–8.92, p= 0.02) and revision THAs (OR: 0.15, CI 95 %: 0.03–0.72, p= 0.02) were associated with postoperative complications. Lower haemoglobin levels (OR: 1.04, CI 95 %: 1.01–1.07, p= 0.004) were associated with higher reoperation risk. **Conclusion**: In a DAIR-treated cohort, increased time from admission to surgery was associated with greater 90 d mortality in THA PJI patients. Timely surgical intervention and optimization of modifiable risk factors are essential to improve outcomes.

## 1 Introduction

In acute prosthetic joint infection (PJI) management, DAIR (debridement, antibiotics, and implant retention) is a commonly used method of treatment. Compared to other treatment modalities, such as single- or two-stage revision, DAIR is associated with less patient morbidity and is more cost-effective and less technically demanding (Szymski et al., 2024). Prior studies have also reported favourable early postoperative outcomes (Grammatopoulos et al., 2017b; Tsang et al., 2017). Nonetheless, treatment failure remains a major concern, particularly due to its association with mortality risk (Toh et al., 2021). Although several patient- and implant-related factors have been reported to influence DAIR outcomes, limited evidence exists regarding modifiable perioperative factors that may impact DAIR in total hip arthroplasty (THA) patients (Tsang et al., 2017; Toh et al., 2021; Wouthuyzen-Bakker et al., 2019, 2020; Espindola et al., 2022).

For several orthopaedic procedures, timing from hospital admission to surgery is a well-established predictor of treatment success. In hip fractures, morbidity and mortality risks are reduced when surgical treatment occurs within 48 h of admission (Papakostidis et al., 2015; Klestil et al., 2018). In acute PJI, recent work in total knee arthroplasty (TKA) demonstrated that treatment delays exceeding 48 h from hospital admission to DAIR are associated with increased septic reoperation rates and postoperative complications (Morgan et al., 2025). However, the impact of timing from admission to DAIR on outcomes in THA PJIs has not been adequately explored. Hence, this study aimed to identify perioperative factors, notably time from admission to surgery, associated with DAIR outcomes in THA PJI.

### 1.1 Methods

#### 1.1.1 Study design and definitions

This retrospective, Institutional Review Board (IRB)-approved study was performed at a high-volume, academic tertiary referral centre specializing in PJI care. Patients having undergone DAIR for acute THA PJIs between 2008 and 2021 were identified from our institutional PJI database. Inclusion criteria consisted of a confirmed acute PJI diagnosis at the time of admission established through clinical examination, laboratory values, and arthrocentesis findings as per the 2018 Musculoskeletal Infection Society (MSIS) criteria (Parvizi et al., 2018). For cases predating 2018 MSIS criteria adoption, retrospective analysis was performed to ensure that the current definition for PJI was met. Acute PJI was defined as a prosthetic hip infection occurring within 6 weeks of the index THA as DAIR has been shown to be effective within this time frame (Terhune et al., 2025; Jacobs et al., 2019). Acute hematogenous infection was defined in accordance with the International Consensus Meeting on Musculoskeletal Infection guidelines as an infection presenting with symptoms of less than 3 weeks' duration following a symptom-free period post-primary index surgery (Bourget-Murray et al., 2024; Chotanaphuti et al., 2019).

Eligible patients underwent DAIR for either primary or revision THA (rTHA) with a minimum 2-year follow-up. In all cases, the decision to proceed with DAIR was made by the treating arthroplasty surgeon at the time of admission. At our institution, DAIR is indicated for acute postoperative or acute hematogenous PJIs occurring within 6 weeks of the index THA or 3 weeks of symptom onset, respectively. PJIs are otherwise managed with one- or two-stage revisions, resection arthroplasty, or amputation, depending on host factors and the infecting organism. During the study period, approximately 51.4 % of the first PJI surgeries were DAIR. Patients were excluded if they were lost to follow-up, underwent previous PJI surgery at an outside institution, had undergone hip resurfacing ( n= 3) or hemiarthroplasty ( n= 28), or had an oncologic prothesis in situ ( n= 1).

#### 1.1.2 Data collection and outcomes

Baseline patient demographic information was recorded, including age, sex at birth, body mass index (BMI), laterality, and American Society of Anaesthesiologist (ASA) classification. Surgical characteristics were also collected, such as type of surgery performed, type of prothesis in situ, indication for index arthroplasty, number of previous hip surgeries, preoperative haemoglobin (HgB), C-reactive protein (CRP) and erythrocyte sedimentation rate (ESR), transfusion count, McPherson host and extremity grades, type of surgery that DAIR was performed on (i.e., primary versus revision), surgical approach at the time of DAIR, preoperative aspirate culture results, intraoperative microbiology culture results, initial and modified antimicrobial regimens, antimicrobial treatment duration, length of hospital stay, PJI chronicity, time from symptom onset to DAIR, time of death, time from admission to DAIR, and time from DAIR to second surgery (Coughlan and Taylor, 2020).

Data collection and analyses were conducted in accordance with the MSIS criteria for successful infection management and standardized outcome reporting guidelines (MSIS ORT) for PJI treatment (Fillingham et al., 2019). PJI treatment success was defined as per the MSIS ORT definition of infection control: tier 1 or tier 2. Tier 1 achieved infection control without ongoing antibiotic therapy, whereas tier 2 achieved infection control with continued suppressive antibiotic therapy.

The primary outcome of this study was reoperation for recalcitrant PJI, whereas secondary outcomes included all-cause 90 d readmission; all-cause 30 and 90 d, and 1-year mortality; use of chronic antibiotic therapy; and postoperative complications as reported by the Clavien–Dindo classification (Dindo et al., 2004). Transfusion requirements were excluded from complication analysis as they were considered to be reflective of patient-specific factors and surgical technique rather than surgical timing (Morgan et al., 2025). Relevant anticoagulation reversal prior to DAIR was performed for all patients, and all but two patients had appropriate reversal (international normalized ratio < 1.5). Analysis was performed to determine the impact of variables, including timing from admission to DAIR, on study outcomes. Subsequent sub-analyses were conducted to identify factors contributing to delays in DAIR > 48 h from admission and to evaluate the impact of treatment delay on antimicrobial duration (Morgan et al., 2025). In addition, a stratified analysis by PJI type was performed, comparing baseline patient demographic information, surgical characteristics, and outcomes of interest in acute early postoperative and acute hematogenous PJIs.

#### 1.1.3 Surgical technique

A formal standardized DAIR protocol was not utilized. Surgical approach, including anterior, lateral, or posterior, was selected by the treating surgeon based on individual case considerations and surgeon preference. Most DAIRs (49 %) were performed through a posterior approach, 41 % through a lateral approach, and 10 % through an anterior approach. All patients underwent comprehensive irrigation and debridement with a minimum of 6 L of normal saline (Abouljoud et al., 2019). The use of additional irrigation solutions was performed at the discretion of the operating surgeon, whereas modular component exchange was performed in all cases.

#### 1.1.4 Microbiology

Five to seven deep tissue samples were obtained intraoperatively at the time of DAIR and cultured for more than 7 d to ensure accurate microbiologic diagnosis (Kheir et al., 2018). Empiric broad-spectrum antibiotics guided by preoperative joint aspiration results were initiated immediately postoperatively, tailored according to intraoperative culture results. Antibiotic regimens, including antibiotic selection and dosing, were adjusted in collaboration with a multidisciplinary team of infectious disease specialists and pharmacists. In the case of negative intraoperative cultures, broad-spectrum antibiotics were adopted following consultation with infectious disease specialists (Bourget-Murray et al., 2024).

#### 1.1.5 Statistical analysis

Descriptive statistics were used to summarize data. Continuous variables were reported as means and standard deviations (SDs). Independent-sample Student's t tests were used to compare means of normally distributed continuous variables, while means of non-normally distributed data were analysed utilizing Mann–Whitney U tests. Categorical variables were compared using $\chi^{2}$ tests. Univariate analyses were performed on the following variables: age, BMI, ASA score, preoperative HgB, CRP and ESR, rTHA, time from admission to DAIR, time from symptom onset to DAIR, and McPherson host and extremity grades. Variables with p<0.1 from the univariate analysis were retained in the multivariate analysis to evaluate their effect on outcomes, including reoperation, readmission, mortality, and complications. The interaction between PJI type and time from admission to DAIR was further assessed in a fixed multivariable model. Results were expressed as odds ratios (ORs) with 95 % confidence intervals (CIs). A p<0.05 was considered to be statistically significant.

## 2 Results

### 2.1 Patient demographics

A total of 100 patients met the inclusion criteria and underwent DAIR for acute PJI treatment (Table 1). The mean age at the time of surgery was 68.8 years ± 13.4 (range 30–100). Of the overall cohort, 55.0 % were female. ASA scores were as follows: 1 ASA I, 10 ASA II, 66 ASA III, and 23 ASA IV.

### 2.2 Surgical characteristics

Most patients underwent DAIR following primary THA ( n= 90) versus rTHA ( n= 10). The mean number of hip surgeries prior to DAIR, including index surgery, was 1.6 (range 1–7). The mean preoperative CRP was 92.4 ± 74.8 mg L^−1^ (range 0.9–249.9), and the mean preoperative ESR was 57.7 ± 31.7 mm h^−1^ (range 2–140). The mean preoperative HgB was 104 ± 17.5 g L^−1^ (range 60–147). The mean length of stay was 28.2 ± 42.9 d. McPherson host and extremity classifications are described in Table 2.

### 2.3 Surgical outcomes for THA PJI patients treated with DAIR

The mean time from admission to DAIR was 46.4 ± 45.1 h (range 3–240), and the timing from admission to surgery was > 48 h in 27.0 % of patients (Table 3). The reasons for delay are demonstrated in Table 4. The mean time from symptom onset to DAIR was 216.7 ± 165.8 h (range 14–504). Following initial DAIR, 54.0 % underwent reoperation, with 52 patients undergoing septic revision for recalcitrant PJI and 2 requiring aseptic revision. Per patient, there was a mean of 1.5 ± 1.8 additional hip surgeries (range 0–10). The mean time from DAIR to second surgery was 199.2 ± 654.3 d (range 3–4395).

**Figure 1:**
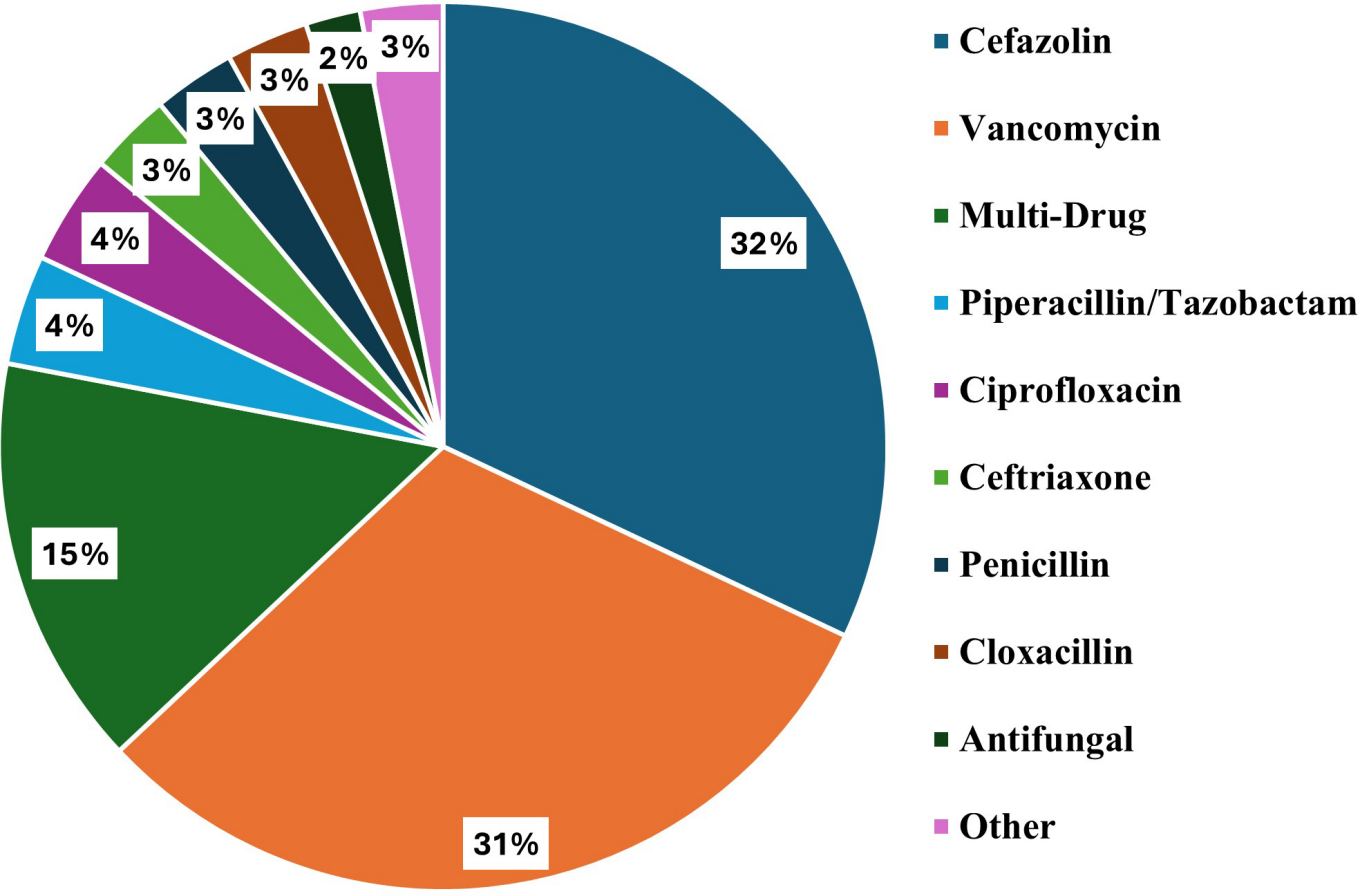
Initial antimicrobial regimens of included patients.

The initial antimicrobial regimens are detailed in Fig. 1. The mean initial therapy duration was 37.0 ± 24.3 d (range 3–109), with 35.0 % of patients requiring modifications based on culture results. As part of ongoing infection management, 42.0 % required chronic suppressive antibiotic therapy post-DAIR. The mean suppressive treatment duration was 729.2 ± 1085.8 d (range 14–4407), with a mean total antimicrobial therapy duration of 333.3 ± 780.9 d (range 3–4447). As shown in Table 5, treatment delays > 48 h were not significantly associated with prolonged duration of initial antimicrobial therapy ( p= 0.71), suppressive treatment ( p= 0.82), or total antimicrobial treatment ( p= 0.52).

**Figure 2:**
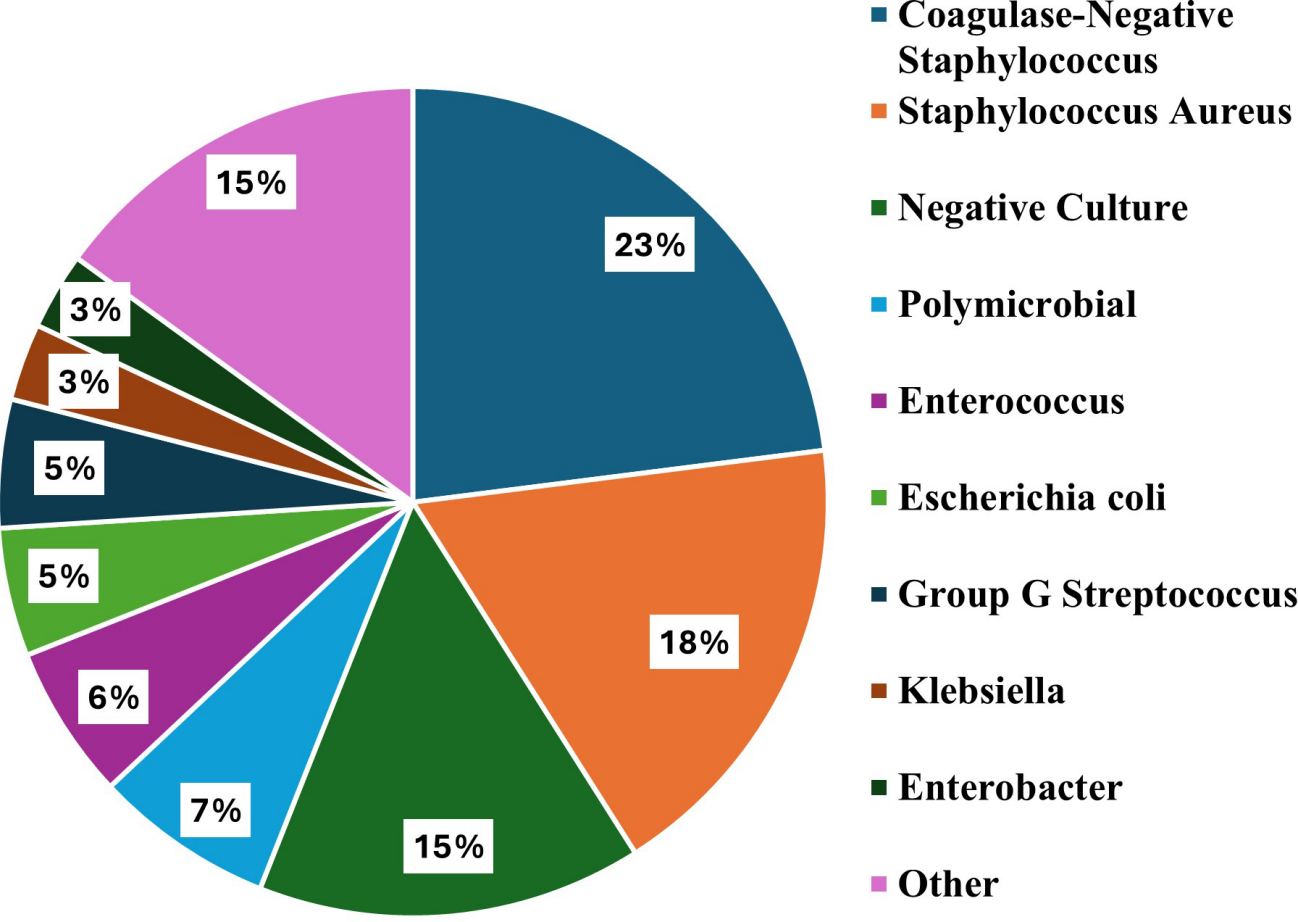
Microorganisms identified intraoperatively at the time of DAIR. DAIR: debridement, antibiotics, and implant retention.

All-cause 90 d readmission occurred in 30.0 % of patients. The rates of 30 and 90 d and 1-year mortality were 3.0 %, 6.0 %, and 12.0 %, respectively. A total of 69.0 % of patients experienced postoperative complications, and, based on the Clavien–Dindo classification, 2.9 % ( n= 2) were grade I, 81.2 % ( n= 56) were grade IIIB, and 15.9 % ( n= 11) were grade V. Microorganisms identified intraoperatively are depicted in Fig. 2. Among patients with a 1-year mortality, 25.0 % had polymicrobial intraoperative cultures, whereas *Enterococcus *and *Enterobacter *species were each identified in 16.7 % of these cases. Intraoperative culture results differed from preoperative aspirates in 22.0 % of patients, while concordant organisms were identified in 49 % of the cohort. Preoperative aspirates were not obtained in 10.0 % of cases, and aspirate data were not available for an additional 19.0 %.

### 2.4 Univariate and multivariate analyses

Table 6 summarizes the univariate analysis, highlighting variables that met the multivariate analysis inclusion threshold, while Table 7 provides an overview of the multivariate analysis. In the multivariate analysis, an increase in time from admission to DAIR by 1 h (OR: 1.02, CI 95 %: 1.00–1.03, p= 0.04) was significantly associated with an increased risk of 90 d mortality. Reoperation was significantly associated with an HgB decrease by 1 g L^−1^ (OR: 1.04, CI 95 %: 1.01–1.07, p= 0.004), and 1-year mortality was significantly associated with an increase in age by 1 year (OR: 1.06, CI 95 %: 1.00–1.12, p= 0.048) and a McPherson host grade C versus A (OR: 12.75, CI 95 %: 1.11–146.09, p= 0.04). An increased risk of postoperative complications was associated with a McPherson host grade C versus A (OR: 7.59, CI 95 %: 1.22–47.08, p= 0.03) and a McPherson extremity grade II versus I (OR: 3.28, CI 95 %: 1.21–8.92, p= 0.02), whereas a decreased risk was linked to rTHA (OR: 0.15, CI 95 %: 0.03–0.72, p= 0.02).

### 2.5 Stratified analysis by infection type

Baseline demographic information for patients with acute early postoperative and acute hematogenous PJI is depicted in Table 8, whereas surgical characteristics for both groups are presented in Table 9. Among all variables, only ASA ( p= 0.01) and preoperative Hgb ( p<0.001 ) differed significantly between groups. In the acute early preoperative group, there were 0 ASA I, 2 ASA II, 38 ASA III, and 6 ASA IV compared to 1 ASA 1, 8 ASA II, 28 ASA III, and 17 ASA IV in the acute hematogenous group. The mean preoperative Hgb was 97.4 ± 14.6 (range 60–130) in the acute early postoperative group and 110.9 ± 17.5 (79–147) in the acute hematogenous PJI group. No statistically significant differences in surgical outcomes were observed by infection type (Table 10). Following interaction testing using a fixed multivariable model, no statistically significant differences were identified for reoperation ( p= 0.99), 90 d readmission ( p= 0.23), 90 d mortality ( p= 0.32), 1-year mortality ( p= 0.09), and postoperative complications ( p= 0.78) (Table 11).

## 3 Discussion

The present study aimed to evaluate the effect of timing from admission to DAIR on treatment outcomes and success in THA patients with acute PJIs. Overall, our findings demonstrate that longer time from admission to DAIR is independently associated with 90 d mortality within this acute PJI cohort. Specifically, multivariate analysis revealed that each additional hour of delay from admission to DAIR conferred, approximately, a 2 % increase in the odds of death, highlighting a potential relationship between earlier surgical intervention and improved outcomes.

A key determinant of successful PJI treatment is the prevention or early disruption of mature biofilm formation (Bourget-Murray et al., 2024). Following deep infections, pathogens establish biofilms on prosthetic surfaces, and, once established, host immune responses and systemic antibiotics become significantly less effective, often requiring component explantation for definitive infection control (Bourget-Murray et al., 2023; Masters et al., 2019; Elkins et al., 2019; Bourget-Murray et al., 2024). Preclinical evidence demonstrates that immature *S. aureus* biofilm formation on implant surfaces can occur as early as 3 h after bacterial attachment, while mature biofilm can form within 24 h (Taha et al., 2023). Although precise timelines for biofilm formation have not yet been translated to the clinical setting, these preclinical data should guide the urgency for prompt PJI surgical management (Nishitani et al., 2015; Elkins et al., 2019; Vidlak and Kielian, 2016; Bourget-Murray et al., 2024). In acute cases, DAIR with modular component exchange generally remains the preferred initial intervention due to its reduced morbidity and favourable outcomes (Bourget-Murray et al., 2024; Grammatopoulos et al., 2017b). However, its success is highly time-sensitive, and efficacy diminishes once biofilm forms on the implant and surrounding tissues (Bourget-Murray et al., 2024). Thus, early DAIR may offer the best opportunity for successful infection control without the need for invasive revision surgery.

Previous research has largely focused on timing from symptom onset to DAIR as a modifiable predictor of THA PJI treatment success. Studies report improved outcomes in THA patients when DAIR is performed within seven days of symptom onset (Tsang et al., 2017; Grammatopoulos et al., 2017a). Others extend this window to 21 d in broader total joint arthroplasty populations (Kunutsor et al., 2018). Conversely, data from Svensson et al. (2020) found no significant differences in THA patients using either time frame, likely due to the inherent subjectivity and variability of patient-reported symptoms. In a similar fashion, our study demonstrated no significant relationship between timing from symptom onset to DAIR and clinical outcomes. Underreporting, late recognition, delayed treatment-seeking behaviour, or contrasting presentations may complicate the utility of symptom duration as a reliable metric for guiding surgical timing in THA-related PJIs. Nevertheless, future work may be merited to identify the underlying causes of delayed presentation, thereby providing actionable targets to optimize treatment timing and outcomes.

On the other hand, timing from admission to surgical intervention offers an objective, quantifiable, and actionable metric. In orthopaedic surgery, timing from admission to surgery has been studied as a predictive factor for treatment success. Research with hip fractures demonstrates that delays > 48 h are linked with increased morbidity, mortality, and length of stays (Klestil et al., 2018; Papakostidis et al., 2015). Similarly, recent work showed that timing from admission to DAIR for TKA PJI > 48 h was associated with higher rates of postoperative complications and reoperation for recalcitrant PJI, suggesting that prompt surgical management is essential (Morgan et al., 2025). In light of these findings, we evaluated the association between timing from admission to DAIR in the THA population and observed similar patterns within this cohort.

To our knowledge, this is the first study to examine the impact of timing from admission to DAIR on outcomes in THA PJIs. Our finding that increased timing from admission to DAIR is associated with increased 90 d mortality adds evidence to support a strong association between prompt surgical intervention and DAIR treatment success.

Notably, the mean time from admission to DAIR in THA PJI patients was approximately 6.6 h longer compared to a previous TKA PJI cohort (Morgan et al., 2025). Among identified reasons for delay, several are modifiable through institutional changes and, therefore, could be minimized or even avoided. For example, operating room availability and optimization of medical comorbidities could be improved through streamlined protocols and better resource allocation. Although withholding direct oral anticoagulants and warfarin for at least 2 and 5 d, respectively, is standard for high-bleed-risk procedures such as THA, time-sensitive cases, like PJI treatment, may benefit from early reversal agent usage (Douketis and Spyropoulos, 2024). Furthermore, delays may, in part, be attributed to the surgeon's comfort level and experience with the procedure and surgical approach (Yan et al., 2023). As such, surgeon availability could have contributed to the longer wait times observed in THA PJI patients. Nonetheless, targeted institutional strategies that address both logistical and provider-related barriers are critical to improving operating room access and optimizing patient outcomes. Identifying these modifiable causes of delay provides an initial framework to guide institutions in developing practical interventions to reduce time to surgery.

Our study further demonstrated a prominent DAIR failure rate, with 52 % of patients returning to the operating room for recalcitrant PJI. Previous studies in THA PJI patients have reported success rates ranging between 58.3 % to 77.5 % (Shaik et al., 2025; Tsang et al., 2017). While timing from admission to surgery is suggested to be a modifiable perioperative risk factor, it is important to note that our institution is a large regional PJI referral centre, resulting in a highly complex patient population and likely contributing to the high reoperation and failure rates observed in this study. Akin to findings demonstrating lower success rates in two-stage revision PJI treatment in a complex patient population, a substantial proportion of patients included in this study presented with significant health-related comorbidities and high-failure-risk causative microorganisms (Tubin et al., 2025). Amongst our THA DAIR cohort, 89.0 % of patients were classified as ASA III or ASA IV, reflecting severe systemic disease, and the average BMI was 31.1 kg m^−2^, categorizing most patients as obese. These factors are independently associated with poorer surgical outcomes and complication rates (Hackett et al., 2015; Madsen et al., 2023). In addition, intraoperative cultures identified a high prevalence of high-failure-risk pathogens, including polymicrobial infections; methicillin-resistant *S. aureus* (MRSA); and gram-negative species like *Enterococcus, Klebsiella*,* Enterobacter, *and *E. coli*, adding considerable complexity to surgical management (Osmon et al., 2013; Zhu et al., 2021; Shohat et al., 2020). Notably, patients with a 1-year mortality more frequently exhibited polymicrobial infections or intraoperative cultures positive for *Enterococcus* or *Enterobacter *species. Consistently with these findings, prior research has reported an increased mortality risk in THA-related PJIs involving *Enterococcus* species (Gundtoft et al., 2017). Thus, our findings underscore the significant multifactorial burden driven by medically and surgically complex patients treated at tertiary centres specializing in PJIs.

In the context of this study, several limitations must be considered. First, the retrospective design inherently introduces selection bias and limits the quality of available data. The accuracy of timing from admission to DAIR may have been affected by a change in electronic medical record systems during the study period, with records lacking complete or consistent documentation. Including both primary and rTHA cases may have introduced additional heterogeneity as the later typically represents a more complex patient population requiring careful consideration before proceeding with DAIR. Nonetheless, this approach allows for a comprehensive assessment of DAIR outcomes across the entire spectrum of THA patients, with these differences adjusted for in the multivariate analyses. However, all relevant confounding variables may not have been captured by our multivariate model. Moreover, the study population comprised both acute early postoperative and acute hematogenous PJI patients. While these groups are classically considered to differ in terms of baseline demographic characteristics and mortality risk, stratified analysis demonstrated significant differences only in ASA classification and preoperative Hgb levels. No differences were observed in the outcomes of interest, including mortality, following statistical analysis and interaction testing between infection type and time from admission to DAIR. In addition, while all cases were treated at the same single tertiary centre specializing in PJIs, variability in DAIR technique and antimicrobial regimens likely existed amongst surgeons and specialists across the 13-year study period. Notably, specialty training in treating PJI is likely to have evolved over time at our centre and may have impacted our results (Tubin et al., 2024). Moreover, we acknowledge the challenges and subjectivity of symptom onset for acute hematogenous PJIs and how this can impact results. However, recent work in DAIR for THA has not demonstrated this to be a significant factor in treatment success (Bourget-Murray et al., 2024). Additionally, implants and surgical materials have also evolved during this time frame, potentially impacting patient outcomes. Furthermore, the decision to proceed with DAIR was ultimately left to the discretion of the on-site attending surgeon. Lastly, several outcomes were associated with wide confidence intervals, reflecting limited precision likely related to the small sample size of the study. Therefore, these findings should be interpreted with caution. Although the findings of this study highlight a linear association between delays in care and outcomes, we were unable to define optimal timing cutoffs for DAIR in THA patients with acute PJIs. Future studies with greater, registry-level patient numbers would be of benefit to more clearly define pragmatic cut-offs for time from admission to DAIR, like what has been done in other areas of orthopaedic surgery.

Overall, this study highlights the impact of the timing of surgery following admission on outcomes following DAIR for acute PJIs in the THA population. Our findings suggest that an increased time from admission to DAIR is associated with greater 90 d mortality within this cohort. These results offer an actionable target to improve treatment success. As such, THA patients with acute PJIs should receive DAIR promptly. Moreover, actions should be taken to minimize potential delays in care. On a system level, institutions should strive to minimize preventable delays and develop clear pathways to facilitate timely operative suite access.

## Data Availability

The data that support the findings of this study are not publicly available due to patient privacy and institutional restrictions but may be made available from the corresponding author upon reasonable request and with appropriate approvals.

## Appendix A Tables

**Table A1:** Baseline demographic information of included patients.

Baseline demographics	N= 100 hips (mean [range ± SD])
Age	68.8 (30–100 ± 13.4)
Sex (female)	55 (55.0 %)
BMI (kg m^−2^)	31.1 (18–51.2 ± 7.8)
Laterality (left)	47 (47.0 %)
ASA Score	
I	1 (1.0 %)
II	10 (10.0 %)
III	66 (66.0 %)
IV	23 (23.0 %)

ASA: American Society of Anesthesiologists; BMI: body mass index; kg: kilogram; m: metre; N : number; SD: standard deviation.

**Table A2:** Surgical characteristics of included patients.

Surgical characteristics	N= 100 hips (mean [range ± SD])
DAIR primary THA versus rTHA	90 (90.0 %)
Indication for index surgery	
OA	67 (67.7 %)
Inflammatory arthritis	4 (4.0 %)
Other	28 (28.3 %)
Preoperative HgB (g L^−1^)	104 (60–147 ± 17.5)
Preoperative CRP (mg L^−1^)	92.4 (0.9–249.9 ± 74.8)
Preoperative ESR (mm h^−1^)	57.5 (2–140 ± 31.7)
RBC transfusion requirements	25 (25.0 %)
LOS (days)	28.2 (3–289 ± 42.9)
Number of surgeries before DAIR, including index	1.6 (1–7 ± 0.99)
Surgical approach	
Anterior	10 (10.0 %)
Lateral	41 (41.0 %)
Posterior	49 (49.0 %)
PJI classification	
Acute early postoperative	46 (46.0 %)
Acute hematogenous	54 (54.0 %)
McPherson host grade	
A	27 (27.0 %)
B	56 (56.0 %)
C	17 (17.0 %)
McPherson extremity grade	
I	48 (48.0 %)
II	52 (52.0 %)

CRP: C-reactive protein; DAIR: debridement, antibiotics, and implant retention; ESR: erythrocyte sedimentation rate; HgB: haemoglobin; LOS: length of stay; N : number; OA: osteoarthritis; PJI: periprosthetic joint infection; rTHA: reverse total hip arthroplasty; SD: standard deviation; THA: total hip arthroplasty.

**Table A3:** Surgical outcomes following THA DAIR in included patients.

Outcomes	N= 100 hips (mean [range ± SD])
Reoperation for PJI	54 (54.0 %)
All-cause readmission within 90 d	30 (30.0 %)
Time from admission to DAIR (hours)	46.4 (3–240 ± 45.1)
30 d mortality	3 (3.0 %)
90 d mortality	6 (6.0 %)
1-year mortality	12 (12.0 %)
Time from symptom onset to DAIR (hours)	216.7 (14–504 ± 165.8)
Postoperative complications	69 (69.0 %)
Clavien–Dindo classification	
I	2 (2.9 %)
IIIB	56 (81.2 %)
V	11 (15.9 %)
Initial antimicrobial therapy duration (days)	37.0 (3–109 ± 24.3)
Therapy modifications after culture results	35 (35.0 %)
Chronic suppressive therapy	42 (42.0 %)
Suppressive treatment duration (days)	729.2 (14–4407 ± 1085.8)
Total antimicrobial therapy duration (days)	333.3 (3–780.9 ± 780.9)
Number of surgeries post-DAIR	1.5 (0–10 ± 1.8)
Time from DAIR to second surgery (days)	199.2 (3–4395 ± 654.3)

DAIR: debridement, antibiotics, and implant retention; N : number; PJI: periprosthetic joint infection; SD: standard deviation.

**Table A4:** Reasons for delay from timing from admission to DAIR > 48 h.

Reason for delay	N= 29 (%)
Unknown/insufficient data	9 (31.0 %)
Optimized medical comorbidities	6 (20.7 %)
Cardiovascular	5 (83.3 %)
Unspecified	1 (16.7 %)
Awaited appropriate anticoagulation reversal	5 (17.2 %)
Operating room availability	4 (13.8 %)
Awaited cultures	4 (13.8 %)
Attempted conservative treatment	1 (3.4 %)

DAIR: debridement, antibiotics, and implant retention; N : number.

**Table A5:** Impact of delay of timing from admission to DAIR > 48 h on duration of antimicrobial therapy.

	Time from admission to DAIR > 48 h (N= 29 Hips)	Time from admission to DAIR ≤ 48 h (N= 71 Hips)	P -Value
Mean initial therapy duration, days (range ± SD)	36.1 (4–92 ± 22.8)	42.9 (3–383 ± 49)	0.71
Mean suppressive therapy duration, days (range ± SD)	996.8 (30–4391 ± 1562.3)	634.2 (14–2977 ± 876.8)	0.82
Mean total duration, days (range ± SD)	379.8 (4–4431 ± 1011.1)	312.7 (3–3033 ± 656.1)	0.52

DAIR: debridement, antibiotics, and implant retention; N : number; SD: standard deviation.

**Table A6:** Univariate analysis of variables with outcomes of interest in included patients.

Outcome	Covariate	Significance
Reoperation	Increase in age by 1 year	OR: 1.00 (CI 95 % 0.97–1.03), p= 0.84
	Obese versus normal BMI	OR: 1.74 (CI 95 % 0.75–4.06), p= 0.20
	ASA 3–4 versus 1–2	OR: 0.98 (CI 95 % 0.28–3.43), p= 0.97
	Increase in preoperative CRP by 1	OR: 1.00 (CI 95 % 1.00–1.01), p= 0.36
	Increase in preoperative ESR by 1	OR: 1.01 (CI 95 % 0.99–1.02), p= 0.23
	Decrease in preoperative HGB by 1^a^	OR: 1.04 (CI 95 % 1.01–1.07), p= 0.004^b^
	Revision THA versus primary THA	OR: 0.33 (CI 95 % 0.08–1.35), p= 0.12
	Increase in time from admission to DAIR by 1 h	OR: 1.00 (CI 95 % 0.99–1.01), p= 0.76
	Increase in time from symptom to DAIR by 1 h	OR: 1.00 (CI 95 % 1.00-1.00), p= 0.15
	McPherson host grade B versus A	OR: 1.24 (CI 95 % 0.50–3.12), p= 0.64
	McPherson host grade C versus A	OR: 1.97 (CI 95 % 0.57–6.88), p= 0.29
	McPherson extremity grade II versus I	OR: 1.36 (CI 95 % 0.62–3.00), p= 0.44
	Acute hematogenous versus acute early preoperative PJI	OR: 0.60 (CI 95 % 0.27–1.32), p= 0.20
90 d readmission	Increase in age by 1 year	OR: 0.98 (CI 95 % 0.95–1.02), p= 0.30
	Obese versus normal BMI	OR: 0.90 (CI 95 % 0.36–2.22), p= 0.82
	ASA 3–4 versus 1–2	OR: 0.72 (CI 95 % 0.19–2.68), p= 0.63
	Increase in preoperative CRP by 1	OR: 1.00 (CI 95 % 0.99–1.00), p= 0.12
	Increase in preoperative ESR by 1	OR: 1.00 (CI 95 % 0.98–1.01), p= 0.54
	Decrease in preoperative HGB by 1	OR: 1.00 (CI 95 % 0.97–1.02), p= 0.76
	Revision THA versus primary THA	OR: 0.23 (CI 95 % 0.03–1.93), p= 0.18
	Increase in time from admission to DAIR by 1 h^a^	OR: 0.99 (CI 95 % 0.97–1.00), p= 0.06
	Increase in time from symptom to DAIR by 1 h	OR: 1.00 (CI 95 % 1.00–1.00), p= 0.94
	McPherson host grade B versus A	OR: 1.59 (CI 95 % 0.57–4.40), p= 0.37
	McPherson host grade C versus A	OR: 0.62 (CI 95 % 0.13–2.79), p= 0.53
	McPherson extremity grade II versus I	OR: 1.31 (CI 95 % 0.55–3.09), p= 0.54
	Acute hematogenous versus acute early preoperative PJI	OR: 0.66 (CI 95 % 0.28–1.55), p= 0.34
90 d Mortality	Increase in age by 1 year^a^	OR: 1.08 (CI 95 % 1.00–1.16), p= 0.051
	Obese versus normal BMI	OR: 3.15 (CI 95 % 0.31–31.48), p= 0.33
	Increase in preoperative CRP by 1^a^	OR: 1.01 (CI 95 % 1.00–1.02), p= 0.08
	Increase in preoperative ESR by 1	OR: 1.01 (CI 95 % 0.99–1.04), p= 0.31
	Decrease in preoperative HGB by 1	OR: 1.04 (CI 95 % 0.98–1.10), p= 0.16
	Increase in time from admission to DAIR by 1 h^a^	OR: 1.01 (CI 95 % 1.00–1.03), p= 0.03^b^
	Increase in time from symptom to DAIR by 1 h	OR: 1.00 (CI 95 % 1.00–1.01), p= 0.88
	McPherson host grade B versus A	OR: 1.47 (CI 95 % 0.15–14.85), p= 0.74
	McPherson host grade C versus A	OR: 3.47 (CI 95 % 0.29–41.53), p= 0.33
	McPherson extremity grade II versus I	OR: 1.92 (CI 95 % 0.33–10.97), p= 0.46
	Acute hematogenous versus acute early preoperative PJI	OR: 0.84 (CI 95 % 0.16–4.39), p= 0.84

**Table A6:** Continued.

Outcome	Covariate	Significance
1-year mortality	Increase in age by 1 year^a^	OR: 1.06 (CI 95 % 1.00–1.12), p= 0.03^b^
	Obese versus normal BMI	OR: 3.32 (CI 95 % 0.63–17.42), p= 0.16
	ASA 3–4 versus 1–2	OR: 1.41 (CI 95 % 0.16–12.10), p= 0.75
	Increase in preoperative CRP by 1	OR: 1.01 (CI 95 % 1.00–1.01), p= 0.23
	Increase in preoperative ESR by 1	OR: 1.01 (CI 95 % 0.99–1.03), p= 0.52
	Decrease in preoperative HGB by 1	OR: 1.01 (CI 95 % 0.98–1.05), p= 0.43
	Revision THA versus primary THA	OR: 0.80 (CI 95 % 0.09–6.92), p= 0.84
	Increase in time from admission to DAIR by 1 h^a^	OR: 1.01 (CI 95 % 1.00–1.02), p= 0.04^b^
	Increase in time from symptom to DAIR by 1 h	OR: 1.00 (CI 95 % 0.99–1.00), p= 0.26
	McPherson host grade B versus A	OR: 3.12 (CI 95 % 0.36–27.31), p= 0.30
	McPherson host grade C versus A^*a*^	OR: 10.83 (CI 95 % 1.14–103.13), p= 0.04^b^
	McPherson extremity grade II versus I	OR: 2.00 (CI 95 % 0.56–7.13), p= 0.29
	Acute hematogenous versus acute early preoperative PJI	OR: 0.83 (CI 95 % 0.25–2.79), p= 0.77
Postoperative complications	Increase in age by 1 year	OR: 1.01 (CI 95 % 0.98–1.04), p= 0.51
	Obese versus normal BMI	OR: 1.52 (CI 95 % 0.62–3.76), p= 0.36
	ASA 3–4 versus 1–2	OR: 1.24 (CI 95 % 0.34–4.60), p= 0.74
	Increase in preoperative CRP by 1	OR: 1.00 (CI 95 % 1.00–1.01), p= 0.53
	Increase in preoperative ESR by 1	OR: 1.01 (CI 95 % 0.99–1.02), p= 0.25
	Decrease in preoperative HGB by 1^a^	OR: 1.02 (CI 95 % 1.00–1.05), p= 0.06
	Revision THA versus primary THA^a^	OR: 0.27 (CI 95 % 0.07–1.04), p= 0.06
	Increase in time from admission to DAIR by 1 h	OR: 1.00 (CI 95 % 0.99–1.01), p= 0.81
	Increase in time from symptom to DAIR by 1 h	OR: 1.00 (CI 95 % 1.00–1.00), p= 0.79
	McPherson host grade B versus A	OR: 2.13 (CI 95 % 0.83–5.49), p= 0.12
	McPherson host grade C versus A^a^	OR: 6.96 (CI 95 % 1.33–36.50), p= 0.02^b^
	McPherson extremity grade II versus I^a^	OR: 2.90 (CI 95 % 1.21–6.96), p= 0.02^b^
	Acute hematogenous versus acute early preoperative PJI	OR: 0.88 (CI 95 % 0.38–2.04), p= 0.76

ASA: American Society of Anesthesiologists; BMI: body mass index; CI: confidence interval; CRP: C-reactive protein; ESR: erythrocyte sedimentation rate; DAIR: debridement, antibiotics, and implant retention; HgB: haemoglobin; OR: odds ratio; THA: total hip arthroplasty. ^a^ Satisfied inclusion for multivariate analysis; ^b^ statistically significant.

**Table A7:** Multivariate Analysis of Variables with Outcomes of Interest in Included Patients.

Outcome	Covariate	Significance
Reoperation	Decrease in preoperative HgB by 1	OR: 1.04 (CI 95 % 1.01–1.07), p= 0.004^*^
90 d readmission	Increase in time from admission to DAIR by 1 h	OR: 0.99 (CI 95 % 0.97–1.00), p= 0.06
90 d mortality	Increase in age by 1 year	OR: 1.08 (CI 95 % 0.98-1.20), p= 0.12
	Increase Preoperative CRP by 1	OR: 1.02 (CI 95 % 1.00–1.03), p= 0.07
	Increase in time from admission to DAIR by 1 h	OR: 1.02 (CI 95 % 1.00–1.03), p= 0.04^*^
1-year mortality	Increase in age by 1 year	OR: 1.06 (CI 95 % 1.00–1.12), p= 0.049^*^
	Increase in time from admission to DAIR by 1 h	OR: 1.01 (CI 95 % 1.00–1.02), p= 0.06
	McPherson host C versus A	OR: 12.75 (CI 95 % 1.11–146.09), p= 0.04^*^
Postoperative complications	Decrease in preoperative HgB by 1	OR: 1.02 (CI 95 % 1.00–1.05), p= 0.10
	Revision versus primary	OR: 0.15 (CI 95 % 0.03–0.72), p= 0.02^*^
	McPherson host C versus A	OR: 7.59 (CI 95 % 1.22-47.08), p= 0.03^*^
	McPherson extremity II versus I	OR: 3.28 (CI 95 % 1.21–8.92), p= 0.02^*^

CI: confidence interval; CRP: C-reactive protein; DAIR: debridement, antibiotics, and implant retention; HgB: haemoglobin; OR: odds ratio. ^*^ Statistically significant.

**Table A8:** Baseline demographic information of included patients by infection type.

Baseline demographics	Acute early postoperative ( N= 46 hips)	Acute hematogenous ( N= 54 hips)	P value
Mean age (range ± SD)	68.0 (38–100 ± 13.1)	69.5 (30–92 ± 13.7)	0.60
Sex, N female	29	26	0.14
Mean BMI, kg m^−2^ (range ± SD)	32.1 (18.0–50.3 ± 8.3)	30.2 (19.4–51.2 ± 7.4)	0.27
Laterality, N left	25	22	0.17
ASA Score, %			0.01^*^
I	0	1	
II	2	8	
III	38	28	
IV	6	17	

ASA: American Society of Anesthesiologists; BMI: body mass index; kg: kilogram; m: metre; N : number; SD: standard deviation.

**Table A9:** Surgical characteristics of included patients by infection type.

Surgical characteristics	Acute early postoperative ( N= 46 Hips)	Acute hematogenous ( N= 54 Hips)	P value
DAIR primary THA versus rTHA, N	43	47	0.34
Indication for index surgery, N			0.71
OA	33	34	
Inflammatory arthritis	2	2	
Other	11	17	
Mean preoperative HgB, g L^−1^ (range ± SD)	97.4 (60–130 ± 14.6)	110.9 (79–147 ± 17.5)	< 0.001^*^
Mean preoperative CRP, mg L^−1^ (range ± SD)	86.5 (0.9–249.9 ± 76.7)	97.6 (1.8–243.7 ± 73.4)	0.47
Mean preoperative ESR, mm h^−1^ (range ± SD)	52.8 (2–126 ± 33.1)	61.8 (11–140 ± 30.1)	0.16
RBC transfusion requirements, N			0.29
0	38	37	
1	5	12	
2	3	5	
Mean LOS, days (range ± SD)	41.8 (3–289 ± 59.2)	16.6 (3–71 ± 13.2)	0.06
Surgical approach, N			0.56
Anterior	6	4	
Lateral	17	24	
Posterior	23	26	
McPherson host grade, N			
A	14	13	
B	25	31	
C	7	10	
McPherson extremity grade, N			0.44
I	24	24	
II	22	30	

CRP: C-reactive protein; DAIR: debridement, antibiotics, and implant retention; ESR: erythrocyte sedimentation rate; HgB: haemoglobin; LOS: length of stay; N : number; OA: osteoarthritis; PJI: periprosthetic joint infection; rTHA: reverse total hip arthroplasty; SD: standard deviation; THA: total hip arthroplasty. ^*^ Statistically significant.

**Table A10:** Surgical outcomes following THA DAIR in included patients by infection type.

Outcomes	Acute early postoperative ( N= 46 Hips)	Acute hematogenous ( N= 54 Hips)	P value
Reoperation for PJI, N	28	26	0.20
All-cause readmission within 90 d, N	16	14	0.34
Mean time from admission to DAIR, hours (range ± SD)	55.2 (4–240 ± 57.4)	38.9 (3–120 ± 29.7)	0.42
30 d mortality, N	1	2	1.00
90 d mortality, N	3	3	1.00
1-year mortality, N	6	6	0.77
Postoperative complications, N	32	36	0.76
Clavien–Dindo classification, N			1.00
I	1	1	
IIIB	26	30	
V	5	6	
Chronic suppressive therapy, N	18	24	0.91
Mean number of surgeries post-DAIR, N (range ± SD)	1.6 (0–10 ± 1.9)	1.3 (0–8 ± 1.7)	0.32

DAIR: debridement, antibiotics, and implant retention; N : number; PJI: periprosthetic joint infection; SD: standard deviation.

**Table A11:** Interaction testing between infection type and time from admission to DAIR using a fixed multivariable model for outcomes of interest.

Outcome	Significance
Reoperation	p= 0.99
90 d readmission	p= 0.23
90 d mortality	p= 0.32
1-year mortality	p= 0.09
Postoperative complications	p= 0.78

DAIR: debridement, antibiotics, and implant retention. ^*^ Statistically significant.

## Code availability

All analyses were conducted using SAS version 9.4 (by SAS Institute Inc., Cary, NC, USA; https://www.sas.com/en_ca/software/sas9.html, last access: 21 March 2026).
